# Oxidative Stress, Maternal Diabetes, and Autism Spectrum Disorders

**DOI:** 10.1155/2018/3717215

**Published:** 2018-11-05

**Authors:** Barbara Carpita, Dario Muti, Liliana Dell'Osso

**Affiliations:** Department of Clinical and Experimental Medicine, University of Pisa, Pisa 55100, Italy

## Abstract

Autism spectrum disorders (ASD) are a group of early-onset neurodevelopmental conditions characterized by alterations in brain connectivity with cascading effects on neuropsychological functions. To date, in the framework of an increasing interest about environmental conditions which could interact with genetic factors in ASD pathogenesis, many authors have stressed that changes in the intrauterine environment at different stages of pregnancy, such as those linked to maternal metabolic pathologies, may lead to long-term conditions in the newborn. In particular, a growing number of epidemiological studies have highlighted the role of obesity and maternal diabetes as a risk factor for developing both somatic and psychiatric disorders in humans, including ASD. While literature still fails in identifying specific etiopathological mechanisms, a growing body of evidence is available about the presence of a relationship between maternal immune dysregulation, inflammation, oxidative stress, and the development of ASD in the offspring. In this framework, results from high-fat diet animal models about the role played by oxidative stress in shaping offspring neurodevelopment may help in clarifying the pathways through which maternal metabolic conditions are linked with ASD. The aim of this review is to provide an overview of literature about the effects of early life insults linked to oxidative stress which may be involved in ASD etiopathogenesis and how this relationship can be explained in biological terms.

## 1. Introduction

Brain development in a fetus and in the first years of life is pivotal in the shaping of the individual overall neuropsychological performance level [1–3]. Any alteration of the intrauterine environment at different stages of pregnancy, such as maternal metabolic pathologies, may lead to long-term condition in the newborn.

Given the above consideration, it should be noted how the rates of obesity and diabetes have experienced a steep increase in several countries, with a correspondent increasing number of studies in literature devoted to this topic [4]. There is a rich literature regarding the effect of excessive weight before pregnancy, particularly in combination with rapid weight gain during pregnancy: a condition generally described as a risk factor for gestational diabetes (GDM), which is glucose intolerance with the onset or first recognition during pregnancy. Up to 15% of pregnant women worldwide are esteemed to be affected from diabetes, and approximately 87.5% of maternal diabetes are GDM. Only 7.5% are preexisting type 1 diabetes [5]. As such, maternal diabetes (and GDM in particular) is an important risk factor for conditions like, but not limited to, miscarriage, macrosomia [6], and neurodevelopmental impairments in the offspring [7–9]. In particular, several studies suggest that the offspring of GDM mothers presents more often language delay, poor motor development, and impaired recognition memory [10–12]. A more specific group of studies focused on the possible link between autism spectrum disorder (ASD) risk in the offspring of GDM mothers [13–18].

ASD is a neurodevelopmental condition characterized by a deficit in social interaction, communication issues, and repetitive, stereotyped behaviors [19–21]. ASD etiopathogenesis is still unclear [22]. However, good evidence for genetic correlates is available: specific genetic mutations can be, in fact, identified in about 20% of ASD cases, and twin studies estimate a heritability between 64 and 91% [23]. This strong genetic influence but the concomitant lack of full concordance in monozygotic twins points to the relevance of environmental factors in the etiopathogenesis of the disorder [24, 25]. Although studies about ASD are focused mainly on children, the presence of this condition is particularly relevant also in adulthood, due to its quite extensive comorbidity with other psychiatric disorders. The presence of undetected ASD among seeking-treatment inpatients is clearly a subject of clinical relevance, highlighting a need for careful investigation of autistic symptoms both in clinical samples and in the general population. A growing body of studies in fact has shown that also the presence of autistic traits (that is, the presence of autism spectrum symptomatology not necessarily in its full-blown clinical presentation) may not only have an impact on increasing the severity of other mental conditions but also be considered as a risk factor for developing other disorders or toward suicidality [26, 27]. Despite that, autistic symptoms often remain undetected in clinical settings, especially in subjects with little cognitive impairments and moderate symptoms, hided by the manifestations of other comorbid disorders [28–31].

The aim of this paper is to review evidence from literature, regarding whether the effects of early life insults and that linked to oxidative stress in particular might be involved in short- and long-term risks for ASD and how this risk can be explained in biological terms.

## 2. Obesity, GDM, and Neurodevelopment: Toward a Comprehensive Model

A good number of studies on rodent or nonhuman primate models focused on the effects of maternal high-fat diet in the offspring. A regime of high-fat diet might vary depending on the studied specimen (mice, rats, and primates) and also on the specific experimental model. As a result, there is a certain degree of variation among these studies. In order to provide a general picture, a common high-fat chow for murine models (“HF”; Research Diets, D12492) provides 60% kcal from fat [32], while primate high-fat food (Test Diet; 5A1F; Purina Mills) provides 32% of calories from fat, and this lab food might be supplemented with calorically dense treats [33]. As reviewed by Sullivan et al. [34], researches on animal models proved that maternal high-fat diet impacts offspring behavior, socialization, and cognition; moreover, it affects reward pathways. Given this data, several studies focused on the biological pathways on such effects, although with controversial results. Most studies on behavioral effects on the offspring of maternal diet have been conducted in rodents. High-fat diet has been shown influencing offspring behavior by modifying maternal care: in particular, by leading to an increased nursing behavior [32, 35]. It has been suggested [34] that this feature might lead to hyperphagia, hypothalamic reprogramming of energy balance-regulating pathways, and—as a result—increased body weight in the offspring. Also, findings from another rodent model show that consumption of high-energy diet in mothers disrupted hippocampal function and thus impaired learning and memory performance [36]. Moreover, maternal high-fat diet has been associated with heightened anxiety in both nonhuman primates and rodents [33, 37]. According to another study, maternal diet rich in polyunsaturated fatty acid produced a highly aggressive offspring, which displayed hyperlocomotion and decreased immobility in a swim test, due probably to an observed protein kinase C downregulation (in the whole brain except the hypothalamus) [38]. High-fat diet is also correlated with alteration in development of cognitive functions. Male offspring from rats exposed to a diet high in saturated or trans fats, once adults, display a deficit in cognitive spatial functioning [37]. Moreover, offspring from obese mothers show a decrease in hippocampal brain-derived neurotrophic factor production (BDNF) which causes an alteration in neurodevelopment for this area [39]. It is also interesting to note how maternal high-fat diet leads to an alteration in reward-based behavior such as food preference in the offspring, as many studies remark [40–43].

One of the most widely reported data about this topic [34] is the fact that high-fat diet consumption seems to expose the offspring to an increase in inflammatory cytokines, which interact with neural development; despite that, only few studies addressed how effects of inflammatory dysregulation can be modulated by the timing and the duration of exposition to such a diet [37].

Sullivan et al. [44] outline two main pathways by which high-fat diet consumption and obesity influence offspring neurodevelopment and subsequently behavior in animal models. Both immune and (neuro-)endocrine systems should be considered affected by this condition. Inflammation plays a clear role, as obesity is associated with elevated inflammatory cytokine production, due to the increase in adipose tissue, to the point that it has been considered as a state of chronic inflammation [45]. Inflammation exposure during gestation is not only associated with perinatal conditions such as premature birth and low birth weight but also associated with neurodevelopmental disorders like ADHD, ASD, and schizophrenia [46–49]. Inflammatory mediators are able to cross the blood-placenta barrier and interact with fetal neurodevelopment. While evidence from rodent models shows that the proinflammatory cytokine interleukin 6 impacts genes for cortical expression [50], it is known that maternal high-fat diet causes a raise of inflammatory markers, with microglial activation and protoinflammatory cytokines in the offspring hippocampus [44].

However, maternal obesity does expose the fetus not only to proinflammatory cytokines but also to an environment where nutrients and neuroendocrine agents like fatty acids, glucose, triglycerides, and leptin are higher. The most immediate effect of this is fetal hyperglycemia. Since glucose can cross the blood-placenta barrier but maternal insulin cannot, the fetus secretes its own insulin, which is also a growth factor involved in brain development. It has been thus hypothesized that hyperinsulinemia in the prenatal period might lead to an alteration in brain development and regulation [51].

Not only insulin but also leptin shows an increased level in obese and diabetic mothers [52]. Leptin receptors are distributed—in humans—among several brain regions which play a central role in behavioral regulation such as the cortex, hippocampus, amygdala, thalamus, and hypothalamus. While linked to inflammatory response [53], leptin is also active in the hypothalamic pituitary adrenal (HPA) axis, a pivotal structure for stress response [44, 54]. As a result, it is interesting to note that higher levels of leptin were detected in ASD children compared to healthy, age-matched controls (Ashwood et al., 2008; [55]).

Leptin leads to consider the role of the HPA axis and its effect on behavior. The HPA axis is responsible for corticotropin-releasing hormone (CRH), and CRH and arginine vasopressin are synthetized in the paraventricular nucleus of the hypothalamus in response to stress. While maternal diet and extension obesity impact both the HPA axis and extrahypothalamic CRH neurons, the interaction of this pathway with neurodevelopment should be addressed [44]. In rat models, high-fat diet in midpregnancy mothers leads to an offspring with increased postnatal basal corticosterone levels in association with reduction of hippocampal and hypothalamic phospholipid-derived arachidonic acid [56]. The Sasaki et al. [57] rat model showed how high-fat diet consumption during pregnancy and lactation led to an offspring with decreased basal corticosterone levels but heightened response to stress with a slower restoration of baseline corticosterone. This offspring also showed an increase in glucocorticoid receptors in the amygdala, with a concurrent alteration in inflammatory gene expression for the hippocampus and amygdala.

In both human and rats, serotonin plays a crucial role for emotional regulation [44]. Inflammation leads to alteration in serotonin regulation, as the animal model described by Ishikawa et al. [58] suggests by showing how rats treated with cytokine-interferon alpha have decreased serotonergic axon density in the amygdala and the ventral medial prefrontal cortex. Offspring from mothers fed a high-fat diet have been reported displaying alteration in the hippocampus, including not only increased 5-HT1A receptors in the ventral hippocampus but also increased brain-derived neurotrophic factor in the dorsal hippocampus (Peleg-Raibstein et al., 2012). A nonhuman primate model showed that maternal high-fat diet consumption impaired the development of the serotonergic system, leading to a reduction of serotonin synthesis and increased anxiety behaviors in the female offspring [33]. This model also shows increased inflammation levels in the hypothalamus among mothers and offspring [33, 44].

Fewer studies investigated also dopamine pathways. A decrease in mesocorticolimbic dopamine sensitivity, with a concurrent decrease in locomotor activation in response to psychostimulant administration, has been found in rat offspring from mothers who have been fed with a high-fat diet during late gestation and lactation [59]. Another study stressed the involvement of genetic expression in this process, showing in a rat that maternal high-fat diet-induced obesity led to dopamine dysregulation in the offspring by the means of genome-wide methylation and gene regulation for dopamine reuptake trasporter, the *μ*-opioid receptor and preproenkephalin [60].

## 3. Maternal Metabolic Conditions and ASD

### 3.1. Oxidative Stress from GDM

As Rossignol and Frye [61] have demonstrated in a large systematic review, strong evidence is available in literature on the links between immune dysregulation, inflammation, oxidative stress, and the etiopathogenesis of autism. The above-considered role of oxidative stress in high-fat diet animal models sheds light, through different pathways, on the widely observed correlation in human population between GDM and poor performance of the offspring on standardized IQ tests and motor development assessments (Rizzo et al., 1997; Ratzon et al., 2000), as well as on abnormalities in the limbic system detected in ASD children samples [18].

To date, many epidemiological studies have proven obesity being a risk factor for development of neuropsychiatric disorders in humans; however, no clear causative link has been yet identified [62].

From an epidemiological point of view, the concomitant rise in prevalence of both obesity and neuropsychiatric disorders has been stressed in literature [63, 64], but it is important to remember how the increase of the latter disorder might be explained with the advancement of diagnostic tools and an improved awareness toward this kind of conditions [65].

In a study on 1004 mothers, diabetes and hypertension were more common among mothers of children affected by ASD and among mothers of children affected by developmental delays (DD) without ASD. Diabetes, in particular, was strongly associated with greater deficits in expressive language in children with ASD and to a less extent to impairment in visual reception, adaptive behavior, motor skills, and receptive/expressive language, as measured by the Social Communication Questionnaire, the Mullen Scales of Early Learning, and the Vineland Adaptive Behavior Scales [18].

Another study with longitudinal design, conducted in a population of 308 mothers with singleton pregnancies, reported different results. Among this population, normal weight women (128) have been divided from overweight (58), obese (52), and GDM ones (76). Among the infants, assessed at 6 and 18 months with the Bayley Scales of Infant Development-III (BSID-III), those born from mothers with pregestational obesity had significantly higher scores in both cognitive and language developments at 6 months of age; however, the authors observe that their absolute numbers are quite low thus suggesting a careful generalization [66].

A study on 2734 mother-child pairs, with an average of 6-year follow-up, found that mothers of children with ASD were significantly more often older and affected by GDM than the mothers of children typically developing (TD). Also, mothers of children with ADHD were more likely to be of lower education, to be obese, and to have used alcohol during pregnancy than the mothers of TD children. It should be noted that since intellectual disabilities (ID) but not other DD showed a pattern of risk increasing with obesity and GDM, the author suggests that ASD with ID may be etiologically distinct from ASD without ID [67].

A large study (*N* = 165311) of mother-child pairs, comprising 17,988 diabetic mothers, shows that there is a significant prevalence, even though the odds ratio is rather low, for ID in children of mothers with diabetes. It is noteworthy that this risk factor is independent of other maternal features like tobacco smoke, ethnicity and race, educational level, birth weight, offspring gender, and hypertension. The authors point out that the link between diabetes in mothers and ID should be identified in maternal and fetal inflammation processes, which poses a risk for abnormal fetal brain development [68]. Furthermore, Wang et al. (2015) found that hypertension related more than pregnancy diabetes with ID in a large (*N* = 123,922) sample of mother-child couples. Huang et al. [69] meta-analysis on this topic shows that maternal diabetes (OR 1.15, *p* < 0.0001) and maternal hypertension, preeclampsia, or eclampsia (OR 1.33, *p* < 0.0001) act as a risk factor toward ID, thus strengthening the hypothesis that oxidative stress- and obesity-caused inflammation can disrupt neurodevelopment.

A similar meta-analysis, this time focusing on ASD, shows that maternal diabetes acts as a risk factor (OR 1.48) without significant heterogeneity (*I*^2^ = 9.1, *p* = 0.35) (Xu et al., 2015). *In utero* exposure to inflammatory factors and hyperglycemia, exempli gratia, could be linked to an increase of free radical production and an impairment of antioxidant countermeasures, through raising the oxidative stress in the cord blood and the placental tissue [70, 71]. These data are even more interesting in the light of studies that take into account the association between maternal autoimmune diseases and subsequent diagnosis of autism in children. A large body of data has been collected on how a family history of autoimmune disorders has been reported more commonly among ASD children than in healthy controls [72, 73]. The Croen et al. [14] case study on 407 couples of mother-children with ASD and 2095 controls has found that maternal autoimmune conditions were significantly associated with ASD in children and asthma in particular. This is coherent with the reported data about midgestation infections by the influenza virus as a risk factor for autism [74, 75], and since the influenza virus cannot cross the placenta, the focus shifts from viral components to the maternal immune response [76–78].

### 3.2. Broadening the Perspective

As above stated, several contributions point out a relationship among GDM, preeclampsia, autoimmune activation, and, in a broader perspective, oxidative stress and inflammation in mothers of ASD children. However, it is clear from these data that disruption to the normal homeostasis of placental environment may increase the risk but not determine psychiatric disorders; thus, the outcome depends also on the other contextual factors which might be involved in the causative algorithm [79]. Maternal inflammatory states and GDM have the peak of their influence during the central part of the gestation [80], when maternal immune activation is also at full efficiency. According to [79], GDM interaction with maternal immune activation might then disrupt the *in utero* environment thus affecting fetal neurodevelopment, as suggested by the role played by interleukin 6 in several animal models [81–83].

Given the role for inflammatory response, oxidative stress and neurodevelopment disruption, it is also useful to point out that eclampsia and also preeclampsia have been considered a risk factor for ASD in many studies. Gardener et al. [84] extensive meta-analysis shows how preeclampsia has been reported being a significant risk factor for ASD, even if the effect size from the various studies is not always consistent. According to Walker et al. [85], preeclampsia is able to affect fetal neurodevelopment by causing an abnormal trophoblast differentiation during embryogenesis and by limiting fetus intake of nutrient and oxygen. This condition primes the syncytiotrophoblast to release proteins into the maternal bloodstream in an attempt to improve circulation. As a side effect, this release might rise baseline systemic inflammation, insulin resistance, and vascular endothelial changes in the mother. The oxidative stress progressive rise is of particular interest, as it could be integrated with the two-stage model of preeclampsia [85, 86]. In this framework, the first state of preeclampsia, characterized by a poorly perfused placenta, is not sufficient to produce the clinical manifestation of preeclampsia. However, this preliminary condition of poor perfusion and oxidative stress interacts with the maternal immune and vascular systems paving the way for the second stage, where the clinical symptoms of preeclampsia occur [86].

In this framework, a potential integration for etiopathogenetic mechanism may come from contribution that takes into account gene interactions and nutritional factors, which might occur at the time of the inflammation, a field which received rising attention from researchers. It has already been pointed out how deficiency of omega-3 fatty acids (n3FAs) might play a decisive role in the etiology of several neurodevelopmental disorders, namely, ASD and ADHD [87–90]. While fish oil supplementation during pregnancy and in the first 3 months after birth leads, at 4-year follow-up, to higher mental age in probands than in controls [91], lower plasma or rbc levels of n3FAs have been found in ASD patients compared to controls [92–94].

According to Field [95], genetic studies outline several connections between n3FA metabolism and neurodevelopmental disorders. Chromosome site 11q22-23, a location linked to ASD and ADHD, contains the genes for desaturases involved in FA conversion, as well as a dopamine receptor gene linked to bipolar disorder. Sites in the 6p21-23 region containing genes involved with fatty acid metabolism are also associated with ASD, ADHD, schizophrenia, and bipolar disorder. The genes for phospholipase A2s, enzymes responsible for transforming Sn-2 long chain fatty acid into free FA molecules, are near genes involved in ADHD, ASD, and bipolar disorder genes [96]. This led several scholars to question how dietary change, as an environmental genetic interaction, might affect etiopathogenesis of neurodevelopmental disorders ([97, 98]; Stevens et al., 1995).

Field [95] proposed to consider how parental age, a risk factor for ASD (less so for ADHD), might be linked to the aging effect on the maternal metabolism of fatty acids, as somatic cells might have defects in delta 6 desaturase activity causing a poorer conversion of fatty acids in older women. Other interesting evidence arises from studies that take into account pregnancy iron deficiency, a more recent trend than the one on fatty acids. Fe is known for its pivotal role in myelination, synaptogenesis, and nerve cell metabolism as a whole; as such, iron deficiency has already been reported as a risk factor for abnormal development While iron deficiency might be associated with various conditions and given the fact that the placenta has an effective regulatory ability for iron transfer, it should be noted that obesity is a risk factor for iron deficiency [1, 3, 99–102]. Berglund et al. [103] noted how this interaction might act as a confounding factor for the link between oxidative stress, inflammation, and neurodevelopment impairments.

Many genetic pathways have been described for autism ([104]; Bae, Hong, 2018), several of which might be connected to the relationship between maternal metabolism, oxidative stress, and neurodevelopment. Of particular interest is the mTOR pathway. A serine-threonine kinase, mTOR (namely, its two protein complexes mTORC1 and mTORC2) is involved in a complex interaction, whose unifying feature could be identified in the integration of multiple intracellular and extracellular signals to coordinate several responses, including protein synthesis, growth, proliferation, and, in the central nervous system, also synaptic plasticity [21, 105]. In particular, mTORC1 and mTORC2 promote the transcription of genes involved in autophage inhibition, protein translation, carbohydrate metabolism, and lipogenesis. As such, they play a central role in the mechanisms of obesity and autoimmune disorders and also of cancer development and aging [106]. The role of mTor has been studied in a large variety of neuropsychiatric disorders, such as mood disorders, schizophrenia, and drug addiction, and it is involved with an alteration in dopaminergic transmission (Ryskalin et al., 2018). Dysregulation of mTOR is a common feature of several neurodevelopmental disorders, such as tuberous sclerosis, which has been associated with ASD in retrospective, prospective, and also meta-analytic studies [107]. Also, Angelman syndrome [108], Rett syndrome [109], CDKL-5 syndrome [110], and Phelan-McDermid syndrome (associated with SHANK 3 deletion and with a high comorbidity with ASD) [21] have been associated with mTOR pathways. This implication of mTOR in autism led to investigating its role as a potential target to understand and treat ASD [111]. Kalkman and Feuerbach [112] argued that ASD syndromes might be caused by mutations in genes that inhibit mTOR and that mTOR might lead to autophagy inhibition. This evidence may shed more light also on the association between allergies, as well as the wide spectrum of autoimmune disorders, and ASD. An interesting study [113] employed BTBR mice, a strain considered a representative model for ASD behavioral deficits [114], that features upregulation of the mTOR-S6K pathway and synaptic inhibition of the mTOR-ULK1 pathway. This animal model has been treated with acute systemic injection of insulin-like growth factor-II, showing an improvement of many major ASD-like behaviors and specifically of cognitive and social deficits, as well as of repetitive behaviors [113].

## 4. Concluding Remarks

In the framework of a growing interest about possible environmental factors involved in the etiopathogenesis of ASD, in particular during *in utero* life, increasing epidemiological reports highlighted the link between ASD and maternal metabolic conditions. Many studies stressed the crucial role for neurodevelopment of the exposition to oxidative stress, which is linked to inflammation, immune dysregulation, and thus, also to a wide variety of metabolic conditions. Despite that, the mechanisms through which oxidative stress might lead to developing ASD remain unclear and not specific, and maternal metabolic conditions have been associated also with different kinds of both somatic and neuropsychiatric disorders in the offspring. Moreover, most of the authors have focused only on singular mechanisms and/or specific metabolic conditions; thus, there is a lack of studies featuring a concomitant evaluation of a broader range of biochemical pathways.

It might be useful to highlight some of the limitations of the present inquiry. This is not a systematic review on the subject, and as such, it bears possible bias under the methodological and interpretative points of view. Moreover, we consider here only some of the many possible ethiopathogenetic paths of autism. As such, the large number of environmental and genetic variables implied in this process has been restricted. In addition, animal models on high-fat diet provide a good picture of some of the mechanisms that might be acting in ASD development, but due to some practical limitations (high-fat or high-energy diet is defined depending on the species), results from these data should be carefully weighted when comparing them to results from humans. Globally, while human clinical conditions such as diabetes mellitus, preeclampsia, or other metabolic conditions may elicit several neurodevelopmental disruptive pathways, it should be noted how these mechanisms, mostly related with oxidative stress, should be better considered in a wider framework of complex interaction with genetic underpinnings as well as with other environmental and neurobiological factors, not limited to the cases here discussed.

Further studies might allow to clarify this perspective, considering in particular the possible multifactorial etiopathogenesis of ASD, which feature the interaction between genetic and environmental conditions. Increasing the knowledge about this topic is of crucial interest for both clinical and research settings, as it may lead not only to improve therapeutic and prevention strategies but also to shed more light on the relationships between central and peripheral systems and between somatic and neuropsychiatric disorders.
